# Dysregulation of miRNAs in Sicilian Patients with Huntington’s Disease

**DOI:** 10.3390/diagnostics15192454

**Published:** 2025-09-25

**Authors:** Michele Salemi, Francesca Antonia Schillaci, Maria Grazia Salluzzo, Giovanna Marchese, Giovanna Maria Ventola, Concetta Simona Perrotta, Vincenzo Di Stefano, Giuseppe Lanza, Raffaele Ferri

**Affiliations:** 1 Oasi Research Institute-IRCCS, 94018 Troina, Italy; fran7.sch@gmail.com (F.A.S.); msalluzzo@oasi.en.it (M.G.S.); glanza@oasi.en.it (G.L.); rferri@oasi.en.it (R.F.); 2 Genomix4Life Srl, 84081 Baronissi, Italy; giovanna.marchese@genomix4life.com (G.M.); giovanna.ventola@genomix4life.com (G.M.V.); 3 Genome Research Center for Health-CRGS, 84081 Baronissi, Italy; 4 Vittorio Emanuele Hospital, Caltanissetta ASP2, 93012 Gela, Italy; kettyperrotta@yahoo.it; 5 Department of Biomedicine, Neuroscience and Advanced Diagnostics (BiND), University of Palermo, 90127 Palermo, Italy; vincenzo19689@gmail.com; 6 Department of Surgery and Medical-Surgical Specialties, University of Catania, 95123 Catania, Italy

**Keywords:** Huntington’s disease, microRNA, neurodegeneration, biomarker, neuroinflammation

## Abstract

**Background/Objectives**: Huntington’s disease (HD) is an autosomal dominant neurodegenerative disorder caused by a CAG nucleotide repeat expansion in the Huntingtin (*HTT*) gene. Dysregulation of microRNAs (miRNAs), key post-transcriptional regulators of gene expression, has been implicated in HD pathogenesis, although their specific roles remain incompletely understood. **Methods**: Peripheral blood mononuclear cells from Sicilian HD patients and matched healthy controls were subjected to small RNA sequencing. Differential expression analysis was conducted using DESeq2 (version 1.44.0), with significance defined as |fold change| ≥ 1.5 and adjusted *p* ≤ 0.05. Ingenuity Pathway Analysis (IPA) was applied to assess functional enrichment, focusing on neurological diseases, inflammatory processes, and miRNA–RNA messenger (mRNA) interaction networks. **Results**: A total of 790 differentially expressed miRNAs were identified in HD patients (270 upregulated and 520 downregulated). IPA revealed enrichment in pathways related to organismal injury, neurological disease, and inflammatory responses. Four major regulatory networks linked differentially expressed miRNAs to neurodegenerative processes, with target genes involved in neuroinflammation, cellular stress responses, and metabolic dysfunction. Cross-referencing with previous RNA-seq data identified 5721 high-confidence miRNA–mRNA interactions, implicating 721 target genes across 54 key canonical pathways. **Conclusions**: HD patients exhibit a distinct and reproducible peripheral blood miRNA expression signature. These dysregulated miRNAs may represent accessible biomarkers and provide mechanistic insights into HD pathogenesis, with potential applications for diagnosis, prognosis, and therapeutic development.

## 1. Introduction

Huntington’s disease (HD) is a late-onset, progressive neurodegenerative disorder, typically manifesting in the second decade of life, with a fatal outcome occurring approximately 6 to 20 years after onset [1,2]. It is an autosomal dominant disease with complete penetrance, meaning that if the trait is inherited, the disease will inevitably develop [2]. The main clinical features of HD include excessive and uncoordinated motor symptoms (chorea), followed by gait disturbances, dystonia, depression, anxiety, and obsessive–compulsive behaviors, all of which progressively worsen throughout the course of the disease. Prior to the manifestation of overt motor symptoms, a prodromal phase is often observed, characterized by subtle motor, cognitive, and behavioral dysfunctions [1,2,3].

In the 20th century, the expansion of the CAG trinucleotide repeat in exon 1 of the huntingtin (*HTT*) gene was identified as the causative factor of the disease [1,4]. This mutation results in the production of a neurotoxic protein containing a stretch of polyglutamine (polyQ) residues near the N-terminus, the length of which is directly proportional to the number of CAG triplet repeats. This leads to neuronal death and neurodegeneration, primarily affecting the striatum and cortex, although the disease ultimately involves both the brain and the body as a whole [1,4,5]. The number of CAG repeats correlates with the age at onset and the rate of disease progression, as well as with the parental origin of the mutated allele [1,2,3]. Specifically, individuals with ≤26 repeats are not considered at risk for HD; alleles with 27–35 repeats are classified as “unstable” and typically exhibit paternal transmission, with offspring often inheriting pathogenic expansions of more than 40 repeats; alleles with 36–39 repeats may or may not lead to HD symptoms at later ages; and alleles with ≥40 repeats are fully penetrant and associated with earlier onset and faster progression [1,2,3]. In approximately 50–70% of cases, there is a direct relationship between CAG repeat length and age of onset, while in the remaining cases, disease expression is thought to be influenced by interactions between modifier genes and environmental factors [3].

Although CAG expansion remains the primary mechanism underlying HD neurodegeneration, in recent years considerable attention has focused on the dysregulation of non-coding RNAs (ncRNAs), particularly microRNAs (miRNAs), which regulate messenger RNA (mRNA) expression by degradation or translational inhibition, both under physiological conditions and in disease states. Since abnormal gene expression underlies many pathologies, miRNAs are believed to influence multiple processes, including growth, development, differentiation, and nervous system functions such as neuronal synthesis, structure, and activity (for details on the synthesis, structure, and function of miRNAs, please refer to the scientific literature) [6,7,8,9].

Studies in knockout animals lacking the Dicer enzyme, essential for miRNA maturation, demonstrated pronounced neuronal deterioration [10,11,12,13]. Zuccato et al. (2003) [14] and Ooi and Wood (2007) [15] identified an interaction between mutant HTT and the Repressor Element 1 Silencing Transcription Factor (REST); under physiological conditions, HTT sequesters REST, but in HD this mechanism fails, leading to pathological *REST* accumulation in neurons. Subsequently, Johnson et al. (2008) [7] showed that elevated REST levels profoundly alter gene expression, in part via miRNA-mediated mechanisms [7,14,15].

Circulating miRNAs in plasma have been proposed as potential biomarkers for monitoring disease progression and for developing patient-specific therapies, based on studies investigating cross-talk between neuronal and peripheral tissues in HD. It has been hypothesized that circulating miRNA profiles are altered by the presence of disease, with general overexpression of miRNAs implicated in metabolic regulation [16]. Furthermore, miRNAs have been measured in cerebrospinal fluid (CSF) from healthy controls, prodromal HD individuals, and patients with manifest disease, revealing six miRNAs that progressively increased in abundance across these groups. These findings suggest that CSF miRNAs may serve as valuable biomarkers for identifying prodromal individuals further from clinical diagnosis, in whom early therapeutic interventions may be more effective and meaningful [17].

Building upon our previous transcriptomic study [18], we conducted a systematic analysis of miRNA expression and performed functional pathway analysis, based on the growing evidence that miRNAs are detectable in multiple extracellular fluids [19]. We also examined miRNA dysregulation in the same HD subjects to explore their potential role as diagnostic, prognostic, and therapeutic biomarkers, thereby hypothesizing a contribution of miRNA alterations to disease development and progression.

## 2. Materials and Methods

### 2.1. Participants

The study enrolled 15 genetically confirmed Sicilian patients with HD (9 males, 6 females; mean age 52.43 ± 15.98 years) and 15 age- and sex-matched healthy controls (11 males, 4 females; mean age 46.33 ± 20.37 years) without neurological, psychiatric, or systemic diseases. All participants were evaluated and diagnosed at the Oasi Research Institute–IRCCS in Troina, Italy. The diagnosis of HD was established on the basis of clinical assessment and molecular testing, confirming a pathogenic CAG repeat expansion (≥40) in the *HTT* gene. Exclusion criteria comprised the presence of other neurodegenerative disorders or any systemic condition at the time of sampling. Written informed consent was obtained from all participants. The study protocol was approved by the Ethics Committee of the Oasi Research Institute–IRCCS in Troina, Italy, on 10 April 2025 (approval code: CEL-IRCCS OASI/10-04-2025/03), and the research was conducted in accordance with the Declaration of Helsinki (1964) and its subsequent amendments.

### 2.2. RNA Extraction

On the same day of collection, the samples were subjected to multiple centrifugation steps to isolate the leukocyte pellet. All pellets were subsequently stored at −80 °C. Total RNA was extracted from PBMCs using TRIzol reagent (Invitrogen Life Technologies, Carlsbad, CA, USA) following the manufacturer’s instructions. Briefly, cells were lysed in TRIzol, phase separation was performed with chloroform, and RNA was precipitated with isopropanol, washed with 75% ethanol, and resuspended in nuclease-free water.

RNA concentration and purity were assessed using the NanoDrop™ 2000/2000c spectrophotometer (Thermo Fisher Scientific, Waltham, MA, USA), while RNA integrity was evaluated with the TapeStation 4200 system (Agilent Technologies, Santa Clara, CA, USA) employing the RNA ScreenTape Assay 2.7. All RNA samples were stored at −80 °C until further processing.

### 2.3. RNA Sequencing and Data Analysis

RNA sequencing and subsequent data analysis were performed by Genomix4Life Srl (Baronissi, Italy). Indexed libraries were prepared from 250 ng of purified RNA using the QIAseq miRNA Library Kit (QIAGEN, Hilden, Germany) according to the manufacturer’s instructions. This kit allows adaptor ligation, reverse transcription and PCR amplification to generate sequencing-ready libraries from low-input RNA samples. Libraries were quantified using the TapeStation 4200 (Agilent Technologies) and the Qubit fluorometer (Thermo Fisher Scientific, Waltham, MA, USA), then pooled to ensure that each index-tagged sample was present in equimolar amounts. The pooled samples underwent cluster generation and sequencing on an Illumina NovaSeq 6000 System (Illumina, Santa Clara, CA, USA) in a 1 × 75 single-end format. Fastq files were subjected to quality control using the FastQC (v0.11.9) tool. The identification and quantification of miRNAs were performed using the library mode of sRNAbench with the Homo sapiens miRBase database (hsa, release 22.1) as reference.

Data normalization and downstream statistical analyses were carried out using the R statistical environment (version 4.4.2). Raw count data from sequencing were normalized using negative binomial generalized linear models (GLMs), considering all genes expressed in at least 30% of the samples to reduce noise from low-abundance features. Differential expression analysis between HD and controls was performed using the DESeq2 package Bioconductor (version 1.44.0), which estimates size factors and dispersion parameters before fitting negative binomial models to identify significantly deregulated miRNAs. miRNAs showing a fold change (FC) ≥ 1.5 or ≤−1.5 ((FC) ≥ 1.5) with adjusted *p*-values (padj) ≤ 0.05, calculated using the Benjamini–Hochberg correction, were considered differentially expressed (DEmiRNAs).

Data visualization was performed in R. Principal component analysis (PCA) and volcano plots were generated using the ggplot2 package (version 3.5.1), while Heatmaps were created using the ComplexHeatmap package (version 2.14.0), applying hierarchical clustering based on Euclidean distance and complete linkage. Functional enrichment analysis was carried out on the set of DEmiRNAs using Ingenuity Pathway Analysis (IPA, QIAGEN). In addition, the IPA microRNA Target Filter was applied to integrate the list of deregulated miRNAs with experimentally validated and predicted mRNA targets, thereby providing insights into downstream regulatory networks (2000–2025 QIAGEN).

The raw sequencing data (fastq files) have been deposited in ArrayExpress under accession number E-MTAB-15153.

## 3. Results

### 3.1. Analysis of Differentially Expressed miRNAs

**Figure 1:**
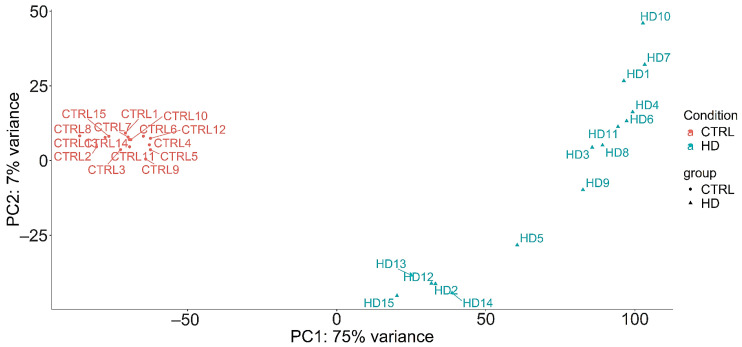
Principal component analysis (PCA) of miRNAs across all samples. Control samples (CTRL) are shown in red, while HD samples are shown in green. The figure displays clustering of the two groups based on the first (PC1) and second (PC2) principal components, which together explain 75% of the total variance.

Small RNA expression profiling was performed using next-generation sequencing in HD patients and matched healthy controls. After removing low-quality reads and trimming adapter sequences, high-quality reads were retained and aligned to the human reference genome. Principal component analysis (PCA) demonstrated a clear clustering pattern, with complete separation between HD patients and control samples, highlighting distinct global small RNA expression profiles between the two groups (Figure 1).

**Figure 2:**
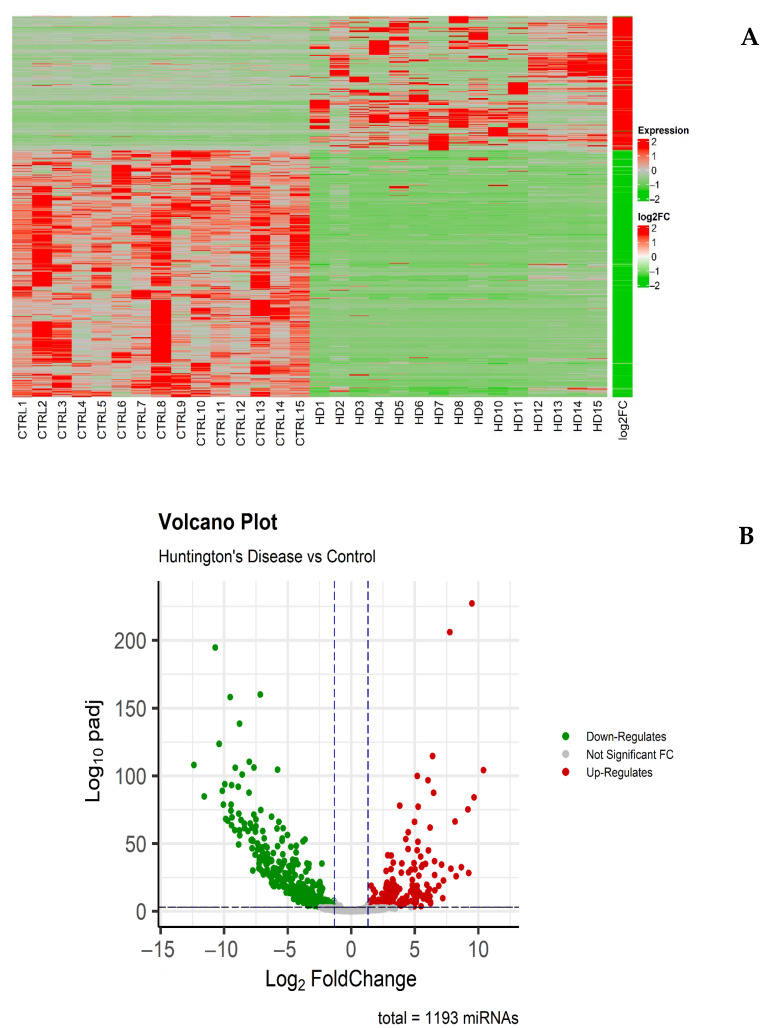
miRNA expression analysis. (**A**) Heatmap of differentially expressed miRNAs in HD versus CTRL samples. Upregulated miRNAs are shown in red and downregulated miRNAs in green; the log2 (fold change) scale bar is also provided. (**B**) Volcano plot displaying the distribution of differentially expressed miRNAs.

To examine the levels and differences in miRNA expression between the two groups (HD vs. CTRL), hierarchical clustering analyses were performed, revealing a total of 1840 unique miRNAs were initially detected across all samples. After quality filtering and expression thresholding, 1193 miRNAs were retained for downstream analysis. Differential expression analysis between HD and control groups identified 790 miRNAs with statistically significant changes (adjusted *p*-value ≤ 0.05), as visualized in the heatmap (Figure 2A) and volcano plot (Figure 2B). Among these, 270 miRNAs were significantly upregulated (fold change ≥ 1.5), while 519 were significantly downregulated (fold change ≤ −1.5), as detailed in Supplementary Table S1. A Core Analysis of these differentially expressed miRNAs (DEmiRNAs) was conducted using IPA, focusing on the Neurological Diseases category.

**Figure 3:**
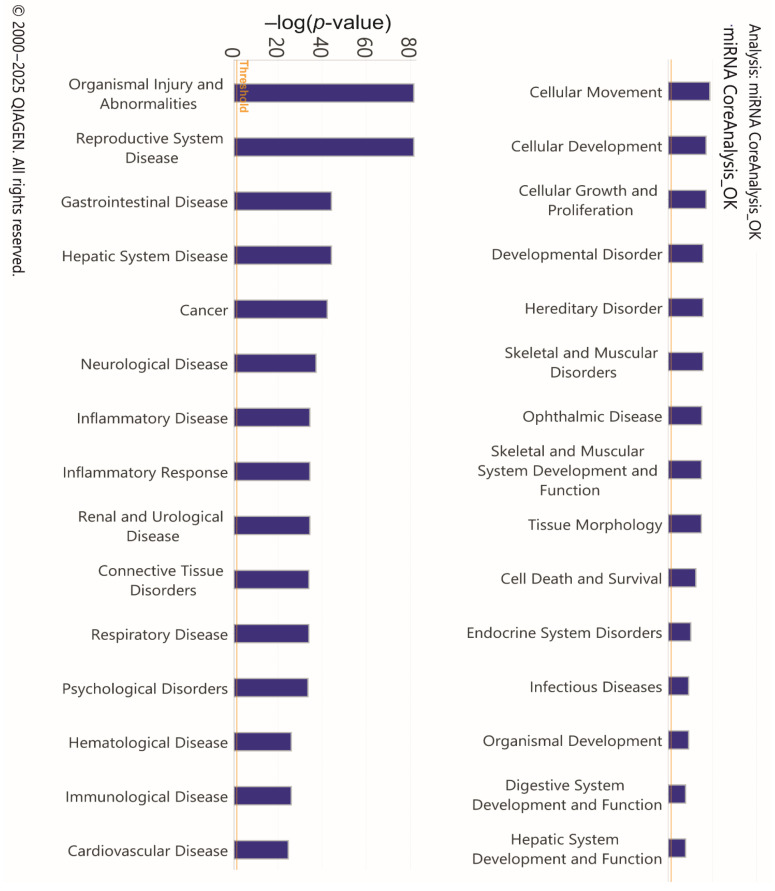
Bar chart of the top 30 Diseases and Bio-Functions identified by IPA. The analysis causally predicts the effects of molecular changes in the dataset on downstream biological processes (“functions”) and diseases. The *y*-axis represents −log(*p*-value).

To investigate the functional involvement of differentially expressed miRNAs and assess their potential association with HD susceptibility, enrichment analysis was performed using IPA. The main analysis identified 62 statistically significant categories within diseases and biological functions, a subset of which is presented in Figure 3 and described in detail in the attached table (Supplementary Table S2). Among the most significantly enriched categories, prominent ones included Organismal Injury and Abnormalities, Neurological Disease, and Inflammatory Response. These represent the principal biological and pathological domains influenced by the analyzed DEmiRNAs, suggesting a potentially important role of these miRNAs in such processes. Although not specific to HD, the categories Inflammatory Response and Organismal Injury and Abnormalities encompass key pathogenic mechanisms such as neuroinflammation, cellular stress, cell cycle dysregulation, and apoptosis, which are well documented in HD neurodegeneration. In particular, the Neurological Disease category was strongly represented, comprising 33 specific annotations related to neurological conditions (Supplementary Table S2). Among these, several are highly relevant to HD, including progressive neurological disorder, progressive encephalopathy, familial motor neuron disease, early symptomatic stage HD, and amyotrophic lateral sclerosis.

**Figure 4:**
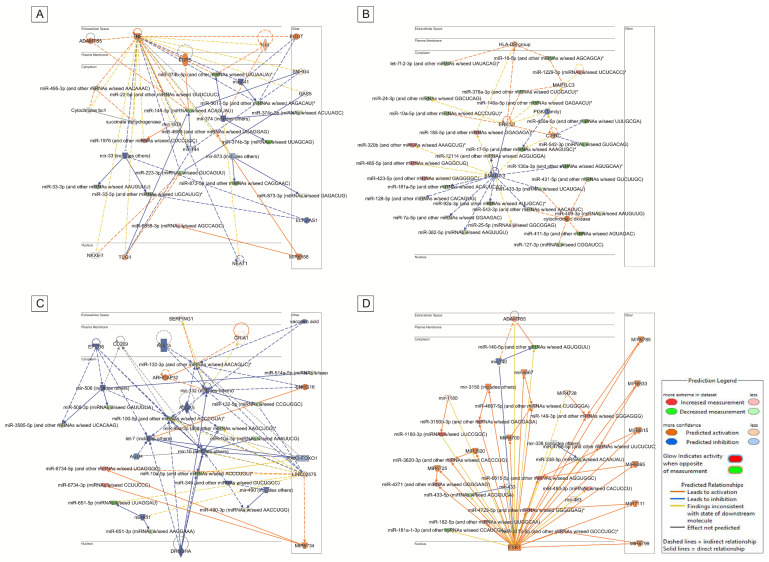
IPA network section: four networks (Network IDs: (**A**) (2), (**B**) (10), (**C**) (18), and (**D**) (22); Supplementary Table S3) associated with neurological pathologies. Coloring is based on the expression values uploaded with the dataset: red indicates upregulation (positive values), green indicates downregulation (negative values), gray indicates molecules present in the dataset but not meeting the user-specified cutoff, and white indicates molecules added from the Ingenuity Knowledge Base. Lines connecting molecules represent molecular relationships. Dashed lines indicate indirect interactions, while solid lines indicate direct interactions. Arrow styles denote the type and directionality of specific molecular relationships. * is the miRNA mature.

Supporting these findings, four networks (Network IDs: 2, 10, 18, and 22) were identified within the Network section of IPA as relevant to neurological pathologies, and they are shown in Figure 4 and (Supplementary Table S3). These networks include genes and DEmiRNAs implicated in neurodegenerative processes, further supporting a potential functional role of these miRNA–gene interactions in neurological diseases. Bioinformatic analysis identified four main interaction networks between miRNAs and their target genes or proteins, distributed across subcellular compartments including the nucleus, cytoplasm, plasma membrane, and extracellular space. These networks, illustrated in panels A–D, display distinct regulatory architectures. Collectively, they underscore the diversity of miRNA-mediated regulatory mechanisms, ranging from centralized control models to distributed systems, highlighting the critical role of miRNAs in modulating gene expression and cellular responses.

### 3.2. Analysis of Target Genes of Differentially Expressed miRNAs

Using IPA, we performed a microRNA Target Filter analysis to investigate the relationships between DEmiRNAs and their putative mRNA targets. This tool integrates both experimentally validated and computationally predicted miRNA–mRNA interactions from the QIAGEN Ingenuity Knowledge Base, enabling prioritization of target genes within the context of our experimental dataset. As RNA-seq analysis had previously been conducted on the same samples, we cross-referenced the 790 DEmiRNAs with the differentially expressed genes (DEGs) identified in the RNA-seq dataset previously published (PIMD accession: E-MTAB-14696).

**Figure 5:**
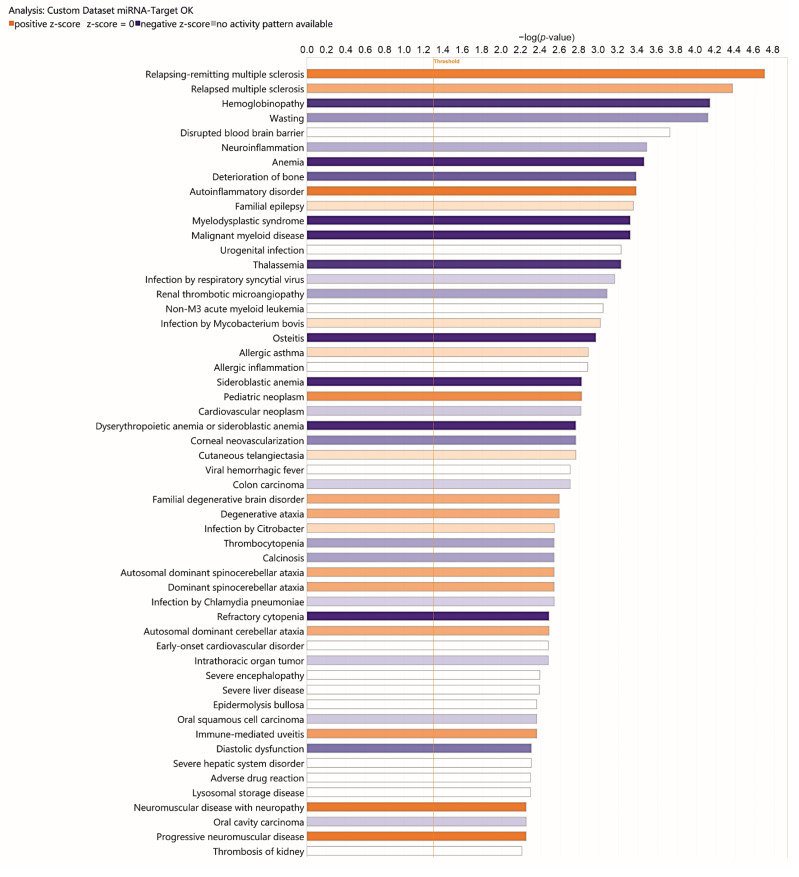
Horizontal bar chart of the top 54 Canonical Pathways identified by Ingenuity Pathway Analysis (IPA) of miRNA target genes. Pathway names are shown on the *y*-axis, and the **x**-axis displays the negative log of the *p*-value, such that taller bars indicate greater significance. Bars are sorted in descending order of significance, with the most enriched pathways at the top of the chart. Orange and purple bars indicate predicted pathway activation or inhibition, respectively, based on the IPA z-score.

**Figure 6:**
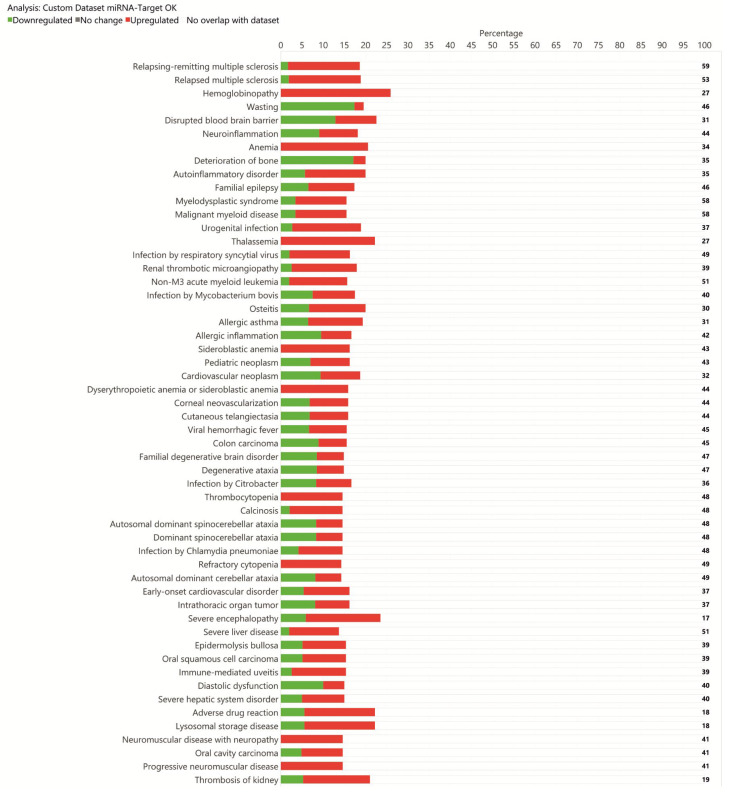
Number of upregulated and downregulated genes involved in each enriched. In the vertical stacked bar chart view, the *x*-axis represents the percentage of molecules present in a specific canonical pathway, while the *y*-axis lists the corresponding molecules.

We selected only miRNA–target interactions that (i) were involved in at least one pathway and (ii) had a confidence score classified as “High-Prediction” and/or “Experimentally Observed” according to IPA (Supplementary Table S4). This filtering process yielded 5721 high-confidence miRNA–mRNA interactions, involving 630 unique miRNAs and 721 target genes. Based on this refined list, we performed a second Core Analysis in IPA, which identified 1381 significantly enriched Canonical Pathways, of which the top 54 were selected for further exploration (see Supplementary Table S5 and Figure 5 and Figure 6).

## 4. Discussion

### 4.1. Main Findings

As shown in Figure 1, PCA demonstrated clear clustering with complete separation between HD patients and control samples, highlighting that differentially expressed miRNAs are representative of each subject belonging to the two groups. This suggests that the miRNA profile of HD subjects is highly specific and reproducible. Figure 2 further demonstrates that differentially expressed miRNAs are almost evenly divided between normal and HD cases. Figure 1 and Figure 2 together confirm that HD is characterized by a highly specific miRNA expression profile, which likely affects various tissues and organs. In this case, the findings specifically demonstrate alterations occurring in peripheral blood leukocytes.

Figure 3 illustrates several of the pathologies and BioFunctions identified through enrichment analysis using IPA. Among these is “Organismal Injury and Abnormalities,” the first bar graph among the 30 principal pathologies and BioFunctions identified. Reported abnormalities in HD subjects include cerebrovascular alterations. In some studies, changes in cerebral blood flow (CBF) have been shown to influence both cognitive status and cerebral vessel density. In two studies on subjects with premotor deficits, CBF alterations were recognized as contributing factors in neurodegenerative disorders and/or as potential diagnostic markers [20,21].

In HD, heart failure has been reported as the second leading cause of death. Studies in mouse models, using instrumental diagnostic techniques, have identified contractile dysfunction and cardiac remodeling (hypertrophy, fibrosis, apoptosis). These phenomena are attributed to nuclear and mitochondrial polyglutamine accumulation in myocytes, as well as increased lysine acetylation and protein nitration, supporting a cardiotoxic effect of mutant HTT [22,23,24,25]. Over several years of research on HD, multiple systemic abnormalities have been identified. However, not all observed alterations have shown a direct and unambiguous correlation with mutant HTT or protein aggregate accumulation in affected tissues and organs. This raises the possibility of additional pathogenic mechanisms that secondarily impact tissues and organs, while ultimately remaining linked upstream to mutant HTT or polyglutamine expansion.

Among the diseases associated with DEmiRNAs in the IPA analysis (Figure 3) are reproductive system disorders. Testicular atrophy is increasingly recognized in HD patients, as demonstrated by postmortem studies showing thickening of seminiferous tubules, impaired spermatogenesis with reduced spermatids and spermatocytes, and disruption of the hypothalamic–pituitary–gonadal axis [26,27,28]. This axis regulates the sequence of events from gonadotropin-releasing hormone (GnRH) secretion to the production of sex hormones (testosterone, estrogen, progesterone) and gametogenesis. Papalexi et al. (2005) developed a mouse model of GnRH expression during sexual maturation, indicating that sexual dysfunction may result from reduced numbers of GnRH-sensitive neurons and diminished GnRH expression [29]. However, no definitive causal factors have yet been established for the sexual alterations observed. To date, the toxic effect of mutant HTT is considered a contributing factor, particularly in relation to reduced testosterone production and impaired neuroblast development [26,27,28,29].

HD is also associated with gastrointestinal disease. Significant weight loss has been consistently reported in HD patients and is considered both a prognostic and predictive marker [30,31,32]. Mutant HTT is ubiquitously expressed, including in the gastrointestinal tract. Altered intestinal motility, diarrhea, and malabsorption have been linked to reduced mucosal thickness, villus elongation, and especially loss of enteric neuropeptides [30,33]. Recent research has increasingly focused on the gut–brain–microbiota axis in HD, investigating potential links between microbiota composition and cognitive, behavioral, and affective function. Wasser et al. (2023) administered probiotics or placebo to 41 HD patients and 36 controls, collecting fecal samples to evaluate clinical effects, but no significant improvements were observed [34,35,36].

Peripheral manifestations of HD also include metabolic and cellular abnormalities (Figure 3) [30,37,38]. Stüwe et al. (2013) demonstrated subclinical hepatic involvement in pre-manifest and manifest HD subjects using the ^13^C-methionine breath test [39]. In mouse models, mHTT aggregates have been identified in the liver, resulting in impaired hepatic function and concomitant downregulation of the peroxisome proliferator-activated receptor gamma (PPARγ). Treatment with PPARγ agonists reduced hepatic mHTT aggregates and improved both liver and central nervous system function [40,41,42]. Comprehensive liver function testing has revealed elevated parameters in manifest HD patients [43], and histological as well as biochemical changes have been described in postmortem liver samples [44]. Together, these findings provide strong evidence for hepatic involvement in HD pathology.

Our IPA results indicate that inflammatory mechanisms play an important role in HD (Supplementary Table S2). Several differentially expressed miRNAs are associated with inflammatory processes, and analysis of their target genes (Supplementary Table S5 and Figure 5) highlighted enrichment in pathways including Neuroinflammation, Autoinflammatory Disorders, Chronic Inflammatory Disorders, and Allergic Inflammation. HD arises from the expansion (>36) of CAG repeats in exon 1 of the HTT gene [45,46], which confers toxic properties on mutant HTT (mHTT), leading to misfolding and aggregation [47,48].

Microglia, discovered as early as 1932, are one of the resident immune cells in the brain [49]. Using specific receptors, they control the microenvironment within the SNC [50,51] and maintain homeostasis through phagocytosis of misfolded proteins, cellular waste, and dying cells [52]. Microglia play a crucial role in supporting the health and function of neurons, and any dysfunction in these cells can have significant consequences in neurodegenerative disorders [51,53].

Following the detection of pathogens or tissue/cell damage, microglia acquire a pro-inflammatory phenotype, characterized by the secretion of pro-inflammatory cytokines such as interleukin (IL)-1β, IL-6, and tumor necrosis factor α (TNFα) [54]. Pro-inflammatory activation of microglia is detectable in HD patients or even at a pre-symptomatic stage [55,56], suggesting that it might be an early event in the pathogenesis of HD; moreover, it correlates with disease progression [57]. It cannot be excluded that this pro-inflammatory state is induced either by the expression of mHTT in microglia or by the response of microglia to the ongoing neurodegenerative process. A study by Steinberg et al. (2023) [58] suggests that the expression of mutant HTT in HD microglia does not result in a heightened microglial response to inflammatory stimuli, but rather causes a cell-autonomous alteration in the development of tolerance that could enable chronic inflammation in the brain and, in turn, contribute to disease progression. Overall, our data are consistent with previous literature and confirm the crucial role of inflammatory mechanisms in HD.

Referring to the IPA analysis (Figure 3), which identified Neurological Diseases, in addition the networks in Figure 4 (see also Supplementary Table S3) include genes and DEmiRNAs implicated in neurodegenerative processes. The bioinformatics analysis identified four main networks of interaction between miRNAs and their target genes, distributed across subcellular compartments including the nucleus, cytoplasm, plasma membrane, and extracellular space. In the top-right panel (Figure 4B), a complex network is observed, organized around several miRNAs that regulate genes involved in signal transduction and cellular stress response. The predominance of blue arrows suggests an overall inhibitory effect of miRNAs on downstream targets, indicating their regulatory role in stress-related pathways. Referring specifically to Supplementary Table S3, from which Figure 4 originates, it is evident that Neurological Diseases are present in various networks, and particularly that network ID 2 shows a score of 46.

Regarding multiple sclerosis (MS), hemoglobinopathy, anemia, and thalassemia, we show that the pathways correlated with relapsing-remitting MS and relapsed MS are also correlated with HD. Referring to Figure 5, both show positive Z-scores, and the target genes of dysregulated miRNAs are upregulated in greater numbers (Figure 5 and Supplementary Table S5). Although the current literature does not show evidence of an association between HD and MS, this does not exclude the possibility that some genes involved in HD, when dysregulated, may affect both diseases. The same data also show that pathways correlated with hemoglobinopathy, anemia, sideroblastic anemia, thrombocytopenia, and refractory cytopenia all have negative Z-scores (Figure 5). In these cases, all target genes of dysregulated miRNAs are upregulated (Figure 5 and Supplementary Table S5). Again, given that the literature does not support associations between HD and blood disorders, it could be hypothesized that neuromuscular impairment leading to inadequate nutrition has repercussions on both hemoglobin levels and hematopoiesis in general, and that as a compensatory response, regulatory mechanisms may be triggered via miRNAs, leading to overexpression of genes involved in these pathways. Finally, we believe that the dysregulation of target genes correlating with Familial Degenerative Brain Disorder and Degenerative Ataxia is of interest (Figure 5).

Evaluating Supplementary Table S6, we show that the least expressed hsa-miR is hsa-miR-590-3p, with a FoldChange of −5355.61. It is known from the literature that higher expression of hsa-miR-590-3p appears to be associated with a higher risk of heart failure [59]. In particular, hsa-miR-590-3p shows regulatory potential by targeting the genes *IL-12B*, *IL-15*, *IL-18*, and *IFNG* [60]; this might correlate with our data highlighting the activation of inflammatory mechanisms. Through bioinformatics analysis, some authors have also shown that hsa-miR-590-3p is involved in cellular senescence and aging of human mesenchymal stem cells [61].

Another noteworthy finding is that miR-590-3p promotes the proliferation, migration, and invasion of pancreatic cancer cells. miR-590-3p directly downregulates p27 and PPP2R2A and, through the G1/S cell cycle pathway, promotes pancreatic cancer development [62]. In this context, it could be hypothesized that hsa-miR-590-3p plays an inverse role in HD and pancreatic ductal carcinoma, contributing to an inverse comorbidity mechanism between the two diseases, as recently reviewed [63].

Hossan et al. (2024) pointed to certain miRNAs, such as hsa-miR-21-5p and hsa-miR-1-3p, hypothesizing that they are involved in transcriptional and post-transcriptional genetic regulation of HD [64]. Rodrigues et al. (2023) [65] evaluated the role of hsa-miR-21-5p in steatohepatitis (NASH)-related liver carcinogenesis and reported that, in both humans and mice, overexpression of this miRNA was detected alongside worsening non-alcoholic fatty liver disease and the formation of pre-neoplastic nodules in mice as the disease progressed. Furthermore, the hsa-miR-21-5p/PPARα pathway was similarly dysregulated in patients with hepatocellular carcinoma (HCC). It was concluded that a therapeutic approach involving inhibition of hsa-miR-21-5p could improve NASH and prevent progression to HCC [65]. Another cancer with high mortality rates, in which the DEmiRNA signature has been studied to identify prognostic biomarkers, is clear cell renal cell carcinoma. Xie et al. (2018) identified a signature of four miRNAs (hsa-miR-21-5p [overexpressed], miR-9-5p, miR-149-5p, and miR-30b-5p [all three underexpressed]) directly correlated with patient survival in clear cell renal cell carcinoma [66]. The same miRNA—hsa-miR-21-5p—overexpressed in individuals with pancreatic cancer, has also been considered a promising serum biomarker in these patients [67].

In a prospective database study, Badacz et al. (2021) [68] selected several miRNAs that could be implicated in the risk of cardiovascular events and followed hundreds of patients for several years. They concluded that the expression of miR-1-3p, miR-16-5p, and miR-122-5p during ischemia may represent a possible risk factor for secondary cardiovascular events [68]. Two of these miRNAs were identified in our analyses and reported in Supplementary Table S6 (hsa-miR-21-5p FoldChange: −1672.97 and hsa-miR-1-3p FoldChange: −3026.73) as downregulated. These two miRNAs have also been identified in other studies in which inverse comorbidity was proposed, whereby miRNAs show opposite expression patterns and are generated by specific genes and pathways that are inversely dysregulated between the two diseases [10,69]. Alternatively, they could reflect comorbidity, which refers to the presence of one or more other diseases in people with an index disease, caused by various factors such as biological, lifestyle, environmental, or medical/therapeutic influences [70].

Lastly, while hsa-miR-4732-3p has not yet been directly investigated in HD, emerging evidence positions it as a neuroprotective and stress-responsive microRNA with potential relevance to neurodegenerative processes. For instance, hsa-miR-4732-3p has demonstrated cardioprotective effects by reducing apoptosis and oxidative stress, promoting angiogenesis, and mitigating fibrosis—particularly within ischemic and doxorubicin-induced injury models [71,72]. Moreover, large-scale cohort studies have identified this miRNA as one of several blood-derived miRNAs associated with cognitive domains and neurodegeneration, suggesting a possible link to brain health and aging processes [73]. Given the hallmark features of HD, including neuronal loss, transcriptional dysregulation, and neuroinflammation, it is plausible that hsa-miR-4732-3p could modulate analogous pathways in HD. Its known roles in anti-apoptotic signaling, angiogenesis, and reactive oxygen species regulation may intersect with the cellular stress responses and neurovascular alterations implicated in HD. Consequently, observing differential expression of hsa-miR-4732-3p in peripheral blood of HD patients may reflect underlying systemic or SNC stress mechanisms with translational relevance.

### 4.2. Limitations

Some limitations should be acknowledged when interpreting the results. First, as is common in research on rare disorders, the sample size was relatively small and limited to Sicilian patients, which may reduce the generalizability of our findings to other populations with different genetic or environmental backgrounds. The limited number of patients and the lack of a targeted and effective therapy for HD do not allow the identification of subgroups using adjuvant medications for psychiatric or movement-related symptoms. Therefore, the case series is not homogeneous from this point of view and cannot be considered an indicator of reproducibility.

Furthermore, although peripheral blood was selected as a minimally invasive source for miRNA profiling, changes in miRNA expression in leukocytes may not fully reflect the molecular alterations occurring in the central nervous system (CNS). Finally, the cross-sectional design precludes conclusions regarding the temporal relationship between miRNA dysregulation and disease onset or progression; longitudinal studies will be required to evaluate the prognostic utility of the identified miRNAs.

## 5. Conclusions

Given that our study is preliminary and based on a modest sample of Sicilian subjects with HD, our data demonstrate a distinct and reproducible miRNA expression profile in the peripheral blood of HD patients, highlighting dysregulated miRNAs involved in pathways associated with neurodegeneration, neuroinflammation, and systemic alterations. Among these, hsa-miR-4732-3p emerges as a potentially relevant candidate warranting further investigation. The identified miRNA–mRNA interaction networks suggest novel mechanistic links that may contribute to biomarker discovery and therapeutic development. Future studies involving larger, longitudinal cohorts and functional validation will be essential to confirm the diagnostic and prognostic potential of these miRNAs and to clarify their role in HD pathophysiology and clinical management. Larger studies to validate the data obtained in this preliminary study are essential.

## Data Availability

Data available in a publicly accessible repository, https://www.ebi.ac.uk/biostudies/ArrayExpress/studies/E-MTAB-15153?query=%20E-MTAB-15153%20.

## Supplementary Materials

The following supporting information can be downloaded at: https://www.mdpi.com/article/10.3390/diagnostics15192454/s1. Table S1: Differentially expressed miRNAs; Table S2: Diseases_and_BioFunctions; Table S3: Networks; Table S4: Def_Mirna-Target_FilterIPA; Table S5: CanonicalPathways_miRNA-Target; Table S6: miRNA_Target.
